# Combined association of triglyceride-glucose index and systolic blood pressure with all-cause and cardiovascular mortality among the general population

**DOI:** 10.1186/s12967-022-03678-z

**Published:** 2022-10-20

**Authors:** Yu Yu, Min Gu, Hao Huang, Sijing Cheng, Yu Deng, Chi Cai, Xuhua Chen, Hongxia Niu, Xiaohui Ning, Wei Hua

**Affiliations:** Cardiac Arrhythmia Center, National Center for Cardiovascular Diseases, State Key Laboratory of Cardiovascular Disease, Fuwai Hospital, Chinese Academy of Medical Sciences and Peking Union MedicalCollege, No. 167 Bei Li Shi Rd, Xicheng District, Beijing, 100037 China

**Keywords:** Triglyceride glucose index, Systolic blood pressure, Mortality, Combined association, National Health and Nutrition Examination Survey

## Abstract

**Background:**

The combined association of triglyceride-glucose (TyG) index and different systolic blood pressure (SBP) levels with all-cause and cardiovascular mortality among the general population remains unclear.

**Methods:**

In this study, 6245 individuals were from the National Health and Nutrition Examination Survey (1999–2002). The study endpoints were all-cause and cardiovascular mortality. Multivariate Cox proportional hazards regression models were used to explore the combined association of TyG index and different SBP levels with all-cause and cardiovascular mortality.

**Results:**

During a mean follow-up period of 66.8 months, a total of 284 all-cause deaths (331/100000 person-years) and 61 cardiovascular deaths (66/100000 person-years) were recorded. Multivariate Cox regression analysis revealed that the combination of low TyG index and low SBP (< 120 mmHg and < 130 mmHg) was associated with a reduced risk of all-cause and cardiovascular mortality than others. However, survival benefit was not observed in the combined group with the low TyG index and SBP < 140 mmHg. Furthermore, the mortality rate in the combined group of low TyG index and low SBP gradually increased with the elevation of SBP level.

**Conclusion:**

The combination of low TyG index and low SBP (< 120 mmHg and < 130 mmHg) was associated with a lower risk of all-cause and cardiovascular mortality. However, no survival benefit was observed in the combined group of low TyG index and SBP < 140 mmHg.

**Supplementary Information:**

The online version contains supplementary material available at 10.1186/s12967-022-03678-z.

## Background

Cardiovascular disease (CVD) and diabetes mellitus (DM) is currently the most prevalent co-morbidities worldwide, and their intrinsic association is based on sharing some common pathological pathways and risk factors [1]. The triglyceride-glucose (TyG) index was regarded as a reliable surrogate biomarker of insulin resistance (IR), first proposed by Simental-Mendía et al. in 2008 [2]. Initial studies found that the TyG index might be a valuable indicator for identifying DM and metabolic syndrome [3, 4]. Subsequently, available studies have reported that the TyG index plays an important role in a variety of CVD, including stable coronary artery disease (CAD), atherosclerosis, coronary artery calcification, acute coronary syndrome (ACS), and stent restenosis [5–10]. On the other hand, hyperglycemia would have deleterious cardiovascular effects. Especially in cancer patients, hyperglycemia could enhance the cardiotoxicity of anticancer agents and attenuate its anticancer efficacy [11]. Apart from the above research evidence, the association between the TyG index and hypertension has rarely been addressed. However, IR and hypertension may develop through a shared pathophysiological pathway that IR triggers excessive activation of the renin–angiotensin–aldosterone system and increases sympathetic nervous system activity [12].

To date, there is consensus that elevated systolic blood pressure (SBP) results in a significantly higher risk of CVD and mortality, but previous evidence suggests that different SBP levels have markedly different effects on prognosis. For example, the Systolic Blood Pressure Intervention Trial (SPRINT) showed that patients with SBP < 120 mmHg could achieve significant cardiovascular and survival benefits compared to the SBP < 140 mmHg group [13]. The recent Strategy of Blood Pressure Intervention in the Elderly Hypertensive Patients (STEP) showed that patients with SBP < 130 mmHg were associated with lower cardiovascular risk than those with SBP < 150 mmHg [14]. In addition to the clinical benefits of lowering blood pressure (BP), it is also critical to consider that the low BP level may affect specific populations. For example, hypotension may be a more significant risk factor than hypertension in patients with small disease-related dementia [15, 16]. The above evidence suggests that patients with different SBP levels would experience a markedly different risk of CVD and mortality, and these differences may significantly impact clinicians' treatment decisions [17]. From this perspective, exploring the combination of the TyG index and different levels of SBP not only fills the gaps in previous studies but also contributes to improved cardiovascular and survival benefits. However, studies on the combined association of the TyG index and different SBP levels on all-cause and cardiovascular mortality are limited.

Therefore, the present study was designed to investigate the combined effect of the TyG index and different SBP levels on mortality and further explore the prevalence of mortality in these combined groups.

## Materials and methods

### Study design and population

NHANES (National Health and Nutrition Examination Survey) is a survey designed to assess adults' and children's health and nutritional status in the United States and is conducted every two years. NHANES uses a complex multistage sampling design, and statistical analysis requires weighting using sample weights to correct for nonresponse and sample design [18]. Data from this study were merged from 2 cycles (1999–2000 and 2001–2002), and a 4-year fasting weight (WTSAF4YR) was applied to the data analysis according to NHANES analysis guidelines. NHANES is conducted by the Centers for Disease Control and Prevention (CDC) and the National Center for Health Statistics (NCHS). The NCHS Research Ethics Review Committee reviewed and approved the NHANES study protocol. All participants signed written informed consent. The NHANES data used in this study can be extracted from DataDryad (https://doi.org/10.5061/dryad.d5h62). The study sample was obtained from the NHANES database from 1999 to 2002. After excluding missing baseline data and individuals under 18 years of age, 6,245 samples entered the final analysis. The specific study population flow chart is shown in Additional file 1: Fig. S1.

### Data collection and definitions

Age, gender, education, physical activity level, smoking, drinking, history of comorbid diseases (hypertension, CAD, and DM), medication history (antihypertensive, statin, and antithrombotic) were collected by questionnaire. Antihypertensive drugs included angiotensin-converting enzyme inhibitors (ACEI), angiotensin receptor blocker (ARB), β-receptor blockers, calcium channel blockers, and diuretics. Antithrombotic drugs include warfarin, aspirin, and clopidogrel. All study participants were measured for height, waist circumference (WC), weight, and heart rate (HR) by trained examiners at the Mobile Examination Center (MEC). Body mass index (BMI) is calculated according to the following formula: BMI = weight (kg)/height (m2). Blood pressure (BP) was the average of at least three measurements taken by two physicians using a standard mercury sphygmomanometer [19]. Fasting venous blood samples were sent to the Lipoprotein Analytical Laboratory (Johns Hopkins University School of Medicine), and total cholesterol (TC), triglycerides (TG), low-density lipoprotein cholesterol (LDL-C), and high-density lipoprotein cholesterol (HDL-C) concentrations were measured using Hitachi 704 Analyzer. Fasting blood glucose (FBG) concentration was measured using a complete blood count (CBC) identification procedure. Glycated hemoglobin (HbA1c) was measured by analyzing whole blood using the Primus Automated HPLC System (Primus I, Model CLC330). Serum creatinine and uric acid (UA) were detected using a Beckman automated clinical analyzer. The estimated glomerular filtration rate (eGFR) was calculated using the abbreviated MDRD formula [20].

### Endpoints and follow-up

The study endpoint was all-cause and cardiovascular mortality. All-cause mortality in this study was mainly caused by heart disease (I00-I09, I11, I13, I20-I51), cerebrovascular disease (I60-I69), cancer (C00-C97) and respiratory disease (J10-J18, J40-J47). Cardiovascular mortality included deaths caused by heart disease (I00-I09, I11, I13, I20-I51) and cerebrovascular disease (I60-I69). The NHANES participants were followed up for mortality through December 31, 2006. Death data were extracted from public-use linked mortality files in the NHANSE database.

### Statistical analysis

All statistical analyses in this study were performed under the guidance of the CDC guidelines (https://wwwn.cdc.gov/nchs/nhanes/tutorials/default.aspx). All statistical analyses of this study were weighted with the sample weights provided by NHANES, as recommended by NCHS. The basic characteristics of the study population are presented with unweighted and weighted samples. For unweighted samples, continuous variables were presented as mean ± standard deviation or median (interquartile range), and categorical variables were presented as proportions. For weighted samples, continuous variables were presented by survey-weighted mean (95% confidence interval (CI)), and categorical variables were presented by survey-weighted percentage (95% CI). Univariate Cox hazard regression analysis was used to explore the association of baseline variables with all-cause and cardiovascular mortality to identify risk factors. The optimal cut-point for the TyG index was determined by receiver operating characteristic (ROC) analysis. Univariate and multivariate Cox hazard regression analyses were used to analyze the combined association of TyG index and SBP with all-cause and cardiovascular mortality. Adjusted variables in multivariate Cox proportional hazards regression models were based on clinical relevance or univariate association with outcomes, and further screening was performed to determine the final regression model based on the number of available events [21]. Bar charts and trend lines were used to show all-cause and cardiovascular mortality trends in the combined variable groups, and mortality rates were calculated using the person-year method. Kaplan–Meier estimator plotted cumulative risk curves for the combined variable groups, and differences between groups were compared using the log-rank test.

All analyses were performed using R statistical software version 4.0.2, and Microsoft Excel (Microsoft, Washington, DC, USA). All P values were 2-sided with a significance level of < 0.05.

## Results

### Baseline characteristics

A total of 6,245 participants were included in the final analysis. The baseline characteristics of the unweighted and weighted samples are presented in Table 1. The mean (95% CI) age of the weighted sample was 42.00 years (41.28, 42.73), and 49.83% were men, of whom 58.16% were at SBP < 120 mmHg, 81.83% were at SBP < 130 mmHg, and 94.92% was at SBP < 140 mmHg). The TyG index and SBP were approximately normally distributed, and the mean (95% CI) of the TyG index and SBP were 8.56 (8.52, 8.59) and 118.64 (117.89, 119.39) mmHg, respectively (Additional file 1: Fig. S2 and S3). Pearson correlation analysis showed a significant positive correlation between the TyG index and SBP (Pearson r = 0.204; p < 0.001), as shown in Additional file 1: Table S1 and Fig. S4.

**Table 1:** Unweighted and weighted baseline characteristics of study population (n = 6245)

Characteristics	Unweighted sample ^a^	Weighted sample ^b^
Age, years	41.78 (18.39)	42.00 (41.28, 42.73)
Male	3031 (48.53%)	49.83% (48.89, 50.77)
BMI, kg/m2	27.80 (6.14)	27.66 (27.38, 27.95)
WC, cm	95.00 (15.50)	94.80 (94.13, 95.47)
SBP, mmHg	119.00 (16.46)	118.64 (117.89, 119.39)
DBP, mmHg	69.74 (13.29)	71.34 (70.72, 71.96)
HR, bpm	72.94 (12.47)	72.45 (71.87, 73.03)
Education		
< High school	2,010 (32.23%)	20.18% (18.32, 22.18)
High school	1,484 (23.80%)	25.16% (23.27, 27.14)
> High school	2,742 (43.97%)	54.66% (51.58, 57.71)
Physical activity		
Sedentary	1,482 (25.57%)	19.13% (17.00, 21.47)
Low	1,558 (26.88%)	27.09% (24.98, 29.32)
Moderate	1,010 (17.43%)	19.83% (18.33, 21.42)
Vigorous	1,746 (30.12%)	33.95% (32.16, 35.78)
Smoking		
Never	2,856 (52.06%)	50.28% (46.64, 53.92)
Former	1,344 (24.50%)	23.74% (21.26, 26.42)
Current	1,286 (23.44%)	25.98% (23.99, 28.07)
Drinking	740 (16.53%)	16.46% (14.91, 18.13)
Diabetes	489 (7.88%)	5.96% (5.14, 6.90)
Hypertension	1,344 (21.72%)	20.41% (18.94, 21.95)
CAD	176 (3.21%)	2.77% (2.28, 3.36)
Antihypertensive drugs	794 (12.71%)	11.11% (10.02, 12.30)
Statins	320 (5.12%)	5.37% (4.58, 6.29)
Antithrombotics	75 (1.20%)	0.91% (0.66, 1.24)
Glucose-lowering drugs	259 (4.15%)	2.91% (2.32, 3.64)
FBG, mmol/L	4.93 (4.59–5.32)	5.19 (5.12, 5.25)
HbA1c, %	5.30 (5.00–5.50)	5.39 (5.34, 5.43)
TC, mmol/L	5.16 (1.13)	5.18 (5.13, 5.23)
TG, mmol/L	1.22 (0.82–1.83)	1.55 (1.49, 1.62)
LDL-C, mmol/L	3.02 (2.45–3.67)	3.12 (3.08, 3.16)
HDL-C, mmol/L	1.34 (0.39)	1.33 (1.31, 1.35)
UA, umol/L	313.17 (88.61)	315.99 (312.86, 319.12)
Creatinine, umol/L	70.72 (53.04–79.56)	74.26 (73.00, 75.52)
eGFR, ml/min/1.73m2	98.02 (69.44–128.03)	106.95 (104.10, 109.81)
TyG index	8.56 (0.68)	8.56 (8.52, 8.59)

*BMI* body mass index, *WC* waist circumference, *SBP* systolic blood pressure, *DBP* diastolic blood pressure, *HR* heart rate, *CAD* coronary atherosclerotic heart disease, *FBG* fasting blood glucose, *HbA1c* glycated haemoglobin, *TC* total cholesterol, *TG* triglycerides, *LDL-C* low-density lipoprotein cholesterol, *HDL-C* high-density lipoprotein cholesterol, *UA* uric acid, *eGFR* estimated glomerular filtration rate, *TyG* triglyceride glucose, *CI* confidence interval

^a^ Data are presented as mean (standard deviation), median (interquartile range) or number (%)

^b^ For continuous variables: survey-weighted mean (95% CI), for categorical Variables: survey-weighted percentage (95% CI)

### Univariate Cox regression analysis of risk factors for all-cause and cardiovascular mortality

During a mean follow-up period of 66.8 months, 284 all-cause deaths occurred, with an all-cause mortality rate of 331 /100000 person-years; 61 cardiovascular deaths occurred, with a cardiovascular mortality rate of 66 /100000 person-years. Univariate Cox regression analysis revealed that age, male, WC, SBP, HR, low education and physical activity level, smoking, drinking, DM, hypertension, CAD, FBG, HbA1c, TC, TG, UA, eGFR, and TyG index were risk factors for all-cause mortality. Meanwhile, age, SBP, low levels of education and physical activity, DM, hypertension, CAD, FBG, HbA1c, TC, UA, eGFR, and TyG index were risk factors for cardiovascular mortality, as shown in Additional file 1: Table S2.

### Combined association of TyG index and different SBP levels with all-cause and cardiovascular mortality

The ROC curves analysis determined that the optimal cut-off points for the predictive value of the TyG index for all-cause and cardiovascular mortality were 8.86 and 8.55, respectively (Additional file 1: Tables S3 and S4). The multivariate-adjusted model in Tables 2, 3 and 4 showed that the low TyG index group was associated with a reduced risk of all-cause and cardiovascular mortality compared with the high TyG index group (all-cause mortality rate: 257/100000 person-years, HR: 0.55, 95% CI 0.39–0.78; cardiovascular mortality rate: 39/100000 person-years, HR: 0.58, 95% CI 0.31–0.96).

**Table 2:** Univariate and multivariate Cox regression analysis of the combined association of TyG index and SBP (120 mmHg) with all-cause and cardiovascular mortality

Variables	Mortality rate (per 100,000 person-years)	Univariate analysis	P-value	Multivariate analysis	P-value
		HR (95% CI)		HR (95% CI)	
All-cause mortality					
TyG category					
≥ 8.86 (High)	518	Ref.		Ref.	
< 8.86 (Low)	257	0.48 (0.37, 0.63)	< 0.001	0.55 (0.39, 0.78)	0.002
SBP category					
≥ 120 mmHg	500	Ref.		Ref.	
< 120 mmHg	209	0.42 (0.31, 0.55)	< 0.001	0.48 (0.34, 0.67)	0.001
Combined variables					
High TyG, SBP ≥ 120 mmHg	655	Ref.		Ref.	
High TyG, SBP < 120 mmHg	361	0.56 (0.36, 0.87)	0.049	0.55 (0.33, 0.91)	0.051
Low TyG, SBP ≥ 120 mmHg	412	0.62 (0.44, 0.87)	0.028	0.62 (0.41, 0.94)	0.023
Low TyG, SBP < 120 mmHg	165	0.25 (0.17, 0.36)	< 0.001	0.29 (0.18, 0.46)	< 0.001
P for trend		< 0.001		< 0.001	
Cardiovascular mortality					
TyG category					
≥ 8.55 (High)	69	Ref.		Ref.	
< 8.55 (Low)	39	0.42 (0.20, 0.88)	0.037	0.58 (0.31, 0.96)	0.044
SBP category					
≥ 120 mmHg	99	Ref.		Ref.	
< 120 mmHg	20	0.17 (0.07, 0.39)	< 0.001	0.28 (0.14, 0.55)	0.001
Combined variables					
High TyG, SBP ≥ 120 mmHg	109	Ref.		Ref.	
High TyG, SBP < 120 mmHg	27	0.23 (0.07, 0.71)	0.021	0.46 (0.19, 1.07)	0.094
Low TyG, SBP ≥ 120 mmHg	86	0.59 (0.26, 1.37)	0.263	0.79 (0.46, 1.97)	0.907
Low TyG, SBP < 120 mmHg	16	0.09 (0.03, 0.29)	< 0.001	0.16 (0.05, 0.46)	< 0.001
P for trend		0.001		0.001	

Model adjusted for age, gender, BMI, physical activity, HR, current smoker, diabetes, antihypertensive drugs, glucose-lowering drugs, LDL-C, HDL-C, UA, eGFR

**Table 3:** Univariate and multivariate Cox regression analysis of the combined association of TyG index and SBP (130 mmHg) with all-cause and cardiovascular mortality

Variables	Mortality ATE (per 100,000 person-years)	Univariate analysis	P-value	Multivariate analysis	
		HR (95% CI)		HR (95% CI)	P-value
All-cause mortality					
TyG category					
≥ 8.86 (High)	518	Ref.		Ref.	
< 8.86 (Low)	257	0.48 (0.37, 0.63)	< 0.001	0.55 (0.39, 0.78)	0.002
SBP category					
≥ 130 mmHg	578	Ref.		Ref.	
< 130 mmHg	276	0.47 (0.35, 0.63)	< 0.001	0.60 (0.43, 0.85)	0.018
Combined variables					
High TyG, SBP ≥ 130 mmHg	671	Ref.		Ref.	
High TyG, SBP < 130 mmHg	465	0.69 (0.44, 1.06)	0.056	0.68 (0.42, 1.10)	0.120
Low TyG, SBP ≥ 130 mmHg	515	0.75 (0.46, 1.22)	0.443	0.64 (0.36, 1.15)	0.211
Low TyG, SBP < 130 mmHg	211	0.30 (0.20, 0.46)	< 0.001	0.36 (0.22, 0.60)	< 0.001
P for trend		< 0.001		< 0.001	
Cardiovascular mortality					
TyG category					
≥ 8.55 (High)	69	Ref.		Ref.	
< 8.55 (Low)	39	0.42 (0.20, 0.88)	0.037	0.58 (0.31, 0.96)	0.044
SBP category					
≥ 130 mmHg	105	Ref.		Ref.	
< 130 mmHg	41	0.35 (0.17, 0.73)	0.003	0.44 (0.23, 0.83)	0.008
Combined variables					
High TyG, SBP ≥ 130 mmHg	109	Ref.		Ref.	
High TyG, SBP < 130 mmHg	56	0.50 (0.20, 1.25)	0.137	0.59 (0.26, 1.36)	0.221
Low TyG, SBP ≥ 130 mmHg	98	0.72 (0.21, 2.48)	0.640	0.83 (0.34, 2.99)	0.992
Low TyG, SBP < 130 mmHg	31	0.19 (0.07, 0.54)	< 0.001	0.32 (0.13, 0.77)	0.003
P for trend		0.001		0.010	

Model adjusted for age, gender, BMI, physical activity, HR, current smoker, diabetes, antihypertensive drugs, glucose-lowering drugs, LDL-C, HDL-C, UA, eGFR

**Table 4:** Univariate and multivariate Cox regression analysis of the combined association of TyG index and SBP (140 mmHg) with all-cause and cardiovascular mortality

Variables	Mortality rate (per 100,000 person-years)	Univariate analysis	P-value	Multivariate analysis	P-value
		HR (95% CI)		HR (95% CI)	
All-cause mortality					
TyG category					
≥ 8.86 (High)	518	Ref.		Ref.	
< 8.86 (Low)	257	0.48 (0.37, 0.63)	< 0.001	0.55 (0.39, 0.78)	0.002
SBP category					
≥ 140 mmHg	512	Ref.		Ref.	
< 140 mmHg	321	0.63 (0.38, 1.05)	0.126	0.75 (0.42, 1.31)	0.354
Combined variables					
High TyG, SBP ≥ 140 mmHg	465	Ref.		Ref.	
High TyG, SBP < 140 mmHg	522	1.16 (0.50, 2.71)	0.678	1.23 (0.48, 3.11)	0.650
Low TyG, SBP ≥ 140 mmHg	541	1.16 (0.42, 3.22)	0.831	1.27 (0.41, 3.91)	0.745
Low TyG, SBP < 140 mmHg	244	0.53 (0.23, 1.22)	0.071	0.62 (0.24, 1.61)	0.261
P for trend		< 0.001		< 0.001	
Cardiovascular mortality					
TyG category					
≥ 8.55 (High)	69	Ref.		Ref.	
< 8.55 (Low)	39	0.42 (0.20, 0.88)	0.037	0.58 (0.31, 0.96)	0.044
SBP category					
≥ 140 mmHg	104	Ref.		Ref.	
< 140 mmHg	50	0.44 (0.14, 1.42)	0.309	0.51 (0.18, 1.43)	0.308
Combined variables					
High TyG, SBP ≥ 140 mmHg	36	Ref.		Ref.	
High TyG, SBP < 140 mmHg	71	1.81 (0.14, 22.90)	0.568	2.75 (0.22, 34.17)	0.326
Low TyG, SBP ≥ 140 mmHg	196	4.14 (0.25, 67.60)	0.298	8.06 (0.54, 121.25)	0.092
Low TyG, SBP < 140 mmHg	33	0.61 (0.05, 8.09)	0.667	0.88 (0.10, 16.29)	0.818
P for trend		0.011		0.029	

Model adjusted for age, gender, BMI, physical activity, HR, current smoker, diabetes, antihypertensive drugs, glucose-lowering drugs, LDL-C, HDL-C, UA, eGFR

**Fig. 1:**
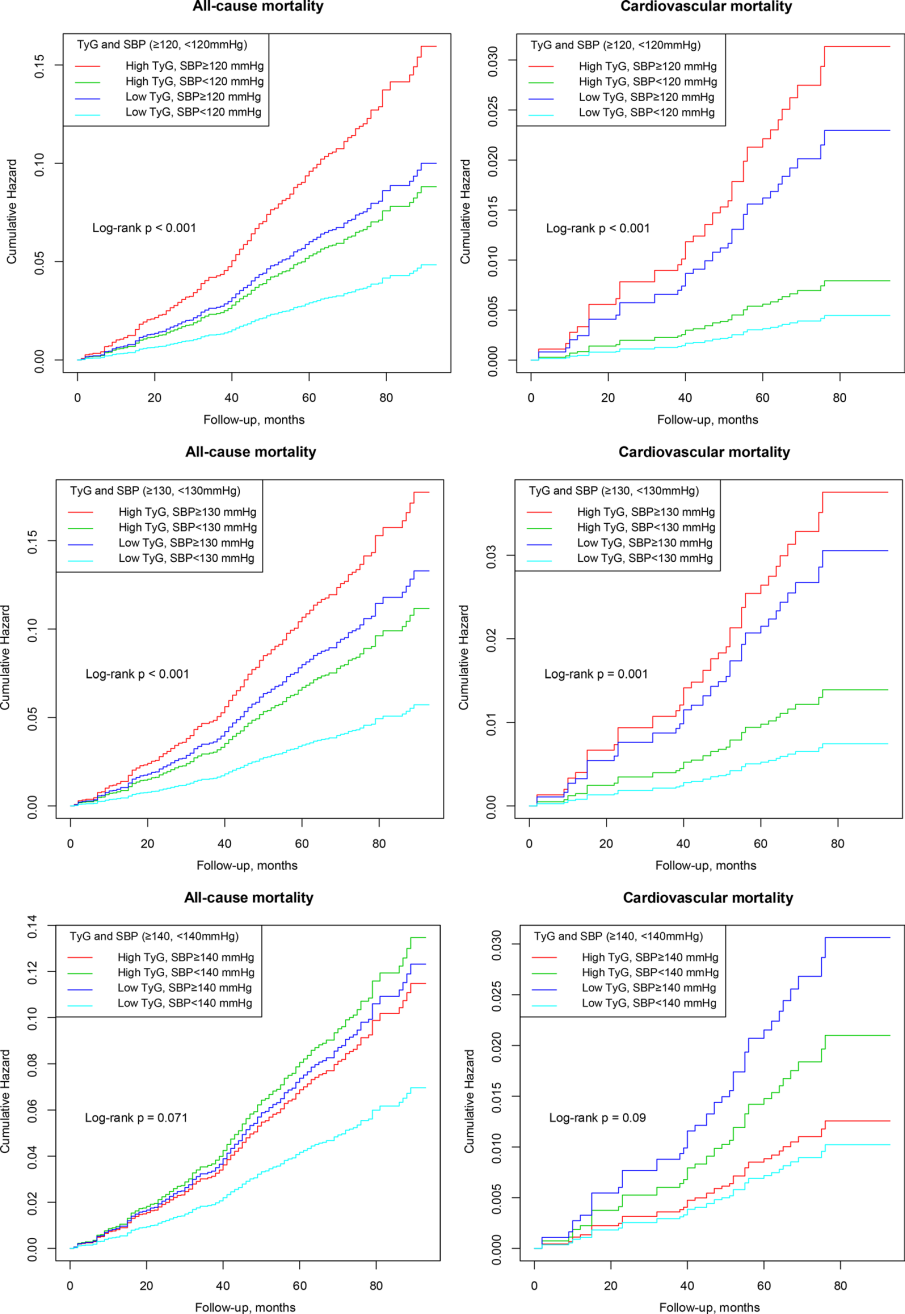
Cumulative incidences of all-cause and cardiovascular mortality by TyG index and different SBP levels

**Fig. 2:**
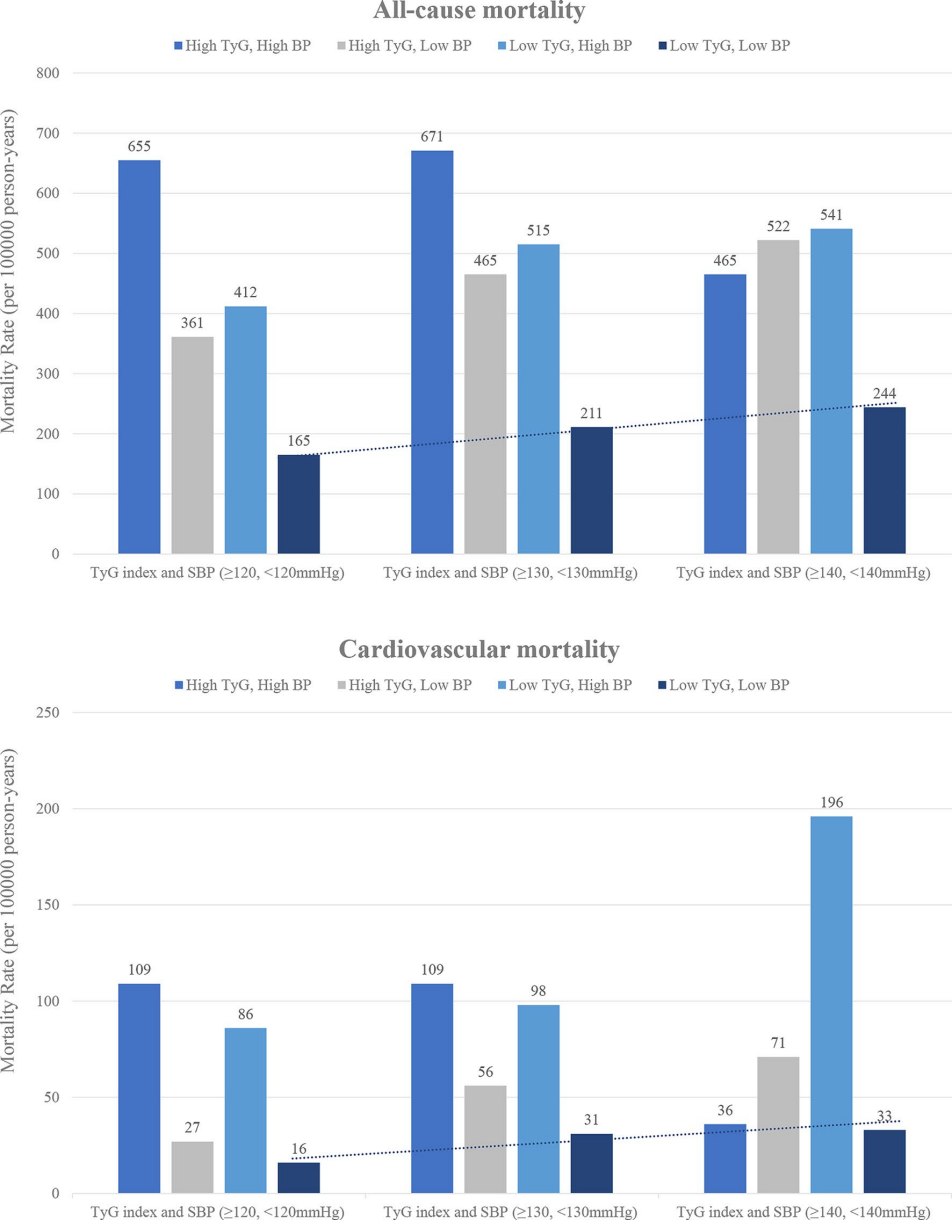
All-cause mortality and cardiovascular mortality in different combination groups and trends

The multivariate-adjusted model in Table 2 showed that the individuals with SBP < 120 mmHg had a lower risk of all-cause and cardiovascular mortality than those with SBP ≥ 120 mmHg (all-cause mortality rate: 209/100000 person-years, HR: 0.48, 95% CI 0.34–0.67; cardiovascular mortality rate: 20/100000 person-years, HR: 0.28, 95% CI 0.14–0.55). The combined group with low TyG index and SBP < 120 mmHg had the lowest risk of all-cause and cardiovascular mortality compared with the other groups (all-cause mortality rate: 165/100000 person-years, HR: 0.29, 95% CI 0.18–0.46; cardiovascular mortality rate: 16/100000 person-years, HR: 0.16, 95% CI 0.05–0.46). In Table 3, the multivariate-adjusted model showed that the individuals with SBP < 130 mmHg were associated with a reduced risk of all-cause and cardiovascular mortality compared with those with SBP ≥ 130 mmHg (all-cause mortality rate: 276/100000 person-years, HR: 0.60, 95% CI 0.43–0.85; cardiovascular mortality rate: 41/100000 person-years, HR: 0.44, 95% CI 0.23–0.83). The combination of low TyG index and SBP ≥ 130 mmHg had the lowest risk of all-cause and cardiovascular mortality compared with the others (all-cause mortality rate: 211/100000 person-years, HR: 0.36, 95% CI 0.22–0.60; cardiovascular mortality rate: 31/100000 person-years, HR: 0.32, 95% CI 0.13–0.77). However, univariate and multivariate analysis showed that the individuals with SBP < 140 mmHg were not different from the SBP ≥ 140 mmHg group in the risk of all-cause and cardiovascular mortality in Table 4. In addition, the combination of low TyG index and SBP < 140 mmHg did not achieve any benefit in all-cause or cardiovascular mortality. During a mean follow-up period of 66.8 months, the combined group with low TyG index and low SBP levels (< 120 mmHg and < 130 mmHg) experienced a lower cumulative risk of all-cause and cardiovascular mortality compared with the other groups (Log-rank p < 0.001, Fig. 1). In addition, the all-cause and cardiovascular mortality in the different combination groups and trends was illustrated in Fig. 2. The low TyG index and SBP < 120 mmHg combination had the lowest all-cause and cardiovascular mortality compared with the other groups. Moreover, all-cause and cardiovascular mortality in the combination of low TyG index and low SBP tended to increase as the level of SBP increased.

## Discussion

Our findings suggest that the TyG index and SBP are risk factors for all-cause and cardiovascular mortality based on a large representative sample of the general US population. The study results showed that low TyG index and SBP levels (< 120 mmHg and < 130 mmHg) were associated with a reduced risk of all-cause and cardiovascular mortality, respectively. More importantly, the combination of low TyG index and low SBP (< 120 mmHg and < 130 mmHg) yielded a further reduction in all-cause and cardiovascular mortality risk than the other groups, with a more significant effect on cardiovascular mortality. However, survival benefit was not observed in the combined group with the low TyG index and SBP < 140 mmHg. Moreover, the mortality rate in the combined group of low TyG index and low SBP gradually increased with the elevation of SBP level.

Currently, growing evidence suggests that the high TyG index is a risk factor for all-cause and cardiovascular mortality in patients with CVD. For example, some studies have identified the TyG index as an independent risk factor for a composite endpoint (with components such as all-cause or cardiovascular death) in patients with stable CAD [5, 22]. Some other studies have shown that the TyG index is an independent predictor of composite endpoints (with components such as all-cause or cardiovascular death) in patients with ACS [6, 23]. Likewise, several studies suggested that the TyG index is a risk factor for all-cause or cardiovascular mortality in patients with HF [10, 24]. However, the above studies only included mortality events as part of the composite endpoint and failed to directly investigate the independent relationship between the TyG index and death. A recent paper revealed a U-shaped relationship between the TyG index and death in hypertensive patients, and this specific relationship may be related to factors such as diet, aging, and low BMI [25]. It is important to note that these findings are from patients with CVD. In fact, the TyG index was calculated from glycemic and triglyceride parameters in healthy individuals [2], so the application of the TyG index in patients with CVD may be influenced by hyperlipidemia or DM, and these confounding factors cannot be eliminated [26]. Indeed, several studies failed to find an independent association between the TyG index and new-onset cardiovascular events in patients with DM or CVD [27, 28]. These results further support that the application of the TyG index in patients with CVD is influenced by hyperlipidemia and DM. The TyG index in this study was derived from healthy individuals, adhering to its initial definition, so that the TyG index can be used to its best value in our study. The findings of this study favorably support the TyG index as a risk factor for all-cause and cardiovascular mortality events in the general population.

As mentioned in the background section, the effect of changes in SBP levels on death is dramatic. The SPRINT study showed that the risk of all-cause and cardiovascular mortality was reduced by 27% and 43% in the group with SBP < 120 mmHg compared to those with SBP < 140 mmHg during a median follow-up of 3.26 years [13]. Due to the tremendous impact of the SPRINT results, the ACC/AHA guidelines subsequently redefined the hypertension diagnostic criteria to ≥ 130/80 mmHg, replacing the previous 140/90 mmHg [29]. The results of the recent STEP study suggest that the patients with SBP < 130 mmHg can make an important contribution to cardiovascular event benefits than those with SBP < 150 mmHg in older patients, but there is no difference in mortality [14]. The above studies suggest different optimal SBP levels, which also provides direction for our study to compare the effect of these SBP levels on mortality in the general population. The results of this study suggest that it is advisable to maintain SBP below 120 or 130 mmHg in the general population. In addition, we conducted a supplementary analysis that SBP at 120–130 mmHg had a significantly higher risk of all-cause and cardiovascular mortality than those with SBP ≤ 120 mmHg. However, when the SBP ≥ 130 mmHg group was used as a reference, individuals with SBP at 120–130 mmHg did not observe a reduction in the risk of all-cause and cardiovascular mortality (Additional file 1: Table S5). This may be because the difference in all-cause and cardiovascular mortality between the groups with SBP at 120–130 mmHg and > 130 mmHg was not significant.

However, long-term hypotension would affect the blood supply to all organs. Especially for vital organs such as the brain, heart, and kidney, insufficient blood supply could trigger related symptoms and organ damage [30]. The available evidence suggests that prolonged hypotension is associated with a higher risk of cognitive decline and vascular dementia [31, 32]. There are three main mechanisms regarding the impaired brain function resulting from hypotension. First, excessive BP reduction would lead to insufficient perfusion of small and medium-sized blood vessels in the brain, which would lead to local brain tissue degeneration and obstruction of cerebral venous return, disrupting the blood–brain barrier [33]. Second, hypotension would weaken the exchange of substances between the cerebrospinal fluid and the brain's interstitial fluid, which prevents the brain from removing metabolic wastes and excess proteins in a timely manner, thereby severely disrupting brain homeostasis [34]. Third, in the case of vascular aging or trauma, the structure and function of cerebral arteries would be damaged to various degrees, resulting in the decline of autoregulation capability [35]. Accordingly, controlled hypotension may also lead to a local insufficiency of blood supply to the brain, greatly increasing the possibility of brain damage. In addition, hypotension could cause insufficient blood supply to the coronary arteries [36]. Especially for patients with CVD, sudden acute episodes of hypotension might induce the occurrence of angina pectoris or even myocardial infarction [37]. Altogether, blindly antihypertensive treatment may not be suitable for all populations. The appropriate lowing BP treatment is worthwhile for the general population. However, for patients with severe cardio-cerebrovascular disease or vascular dementia, hypotension may bring them potential risks, so the blind pursuit of low BP levels requires careful consideration.

The introduction of the TyG index and SBP as combined variables into our study is based on two main aspects. On the one hand, the TyG index and SBP are risk factors for CVD and mortality [38, 39]. It seems reasonable that combining these two risk factors is supposed to generate a cumulative risk of mortality, but this hypothesis has not been confirmed in previous studies. The findings confirm our hypothesis that the combination of low TyG index and low SBP (< 120 mmHg and < 130 mmHg) resulted in an incremental effect on reducing all-cause and cardiovascular mortality risk. Furthermore, the combined TyG index and SBP can greatly facilitate the application of the TyG index in clinical treatment. A clinician will usually pay attention to BP values first in analyzing a patient's clinical data, and the TyG index does not appear directly on the laboratory test orders. This study suggests that it is advisable for clinicians to monitor adverse changes in BP, blood lipids, and FBG simultaneously during clinical treatment. Notably, the TyG index may vary with changes in BP levels in individuals. Therefore, we used Pearson correlation analysis to examine the correlation between the TyG index and SBP. The results showed that the correlation between SBP and TyG index was significant (p < 0.0001), but r < 0.3 indicated the correlation was very weak (Additional file 1: Fig. S4 and Table S1). Therefore, the bias from the interaction of the two may not influence the study results significantly.

The results of this study suggest that SBP may have a more important diagnostic value than the TyG index for all-cause and cardiovascular mortality. Therefore, we performed a ROC analysis to compare the diagnostic value of SBP and TyG index for all-cause and cardiovascular mortality. The results showed that SBP had a more important determinant value for all-cause (AUC: 0.630 vs. 0.604) and cardiovascular mortality (AUC: 0.665 vs. 0.570) than the TyG index (Additional file 1: Fig. S5). An important finding in this study was that elevated SBP (< 140 mmHg) combined with low TyG index did not yield a survival benefit. In contrast, the SBP < 140 mmHg group was only 10 mmHg higher than the SBP < 130 mmHg group. This phenomenon may be attributed to the significantly higher risk of all-cause and cardiovascular mortality in the population at 130–140 mmHg, with these increased risks offsetting the survival benefit of SBP < 140 mmHg. As shown in the Additional file 1: Table S6, the 130–140 mmHg group had the highest risk of all-cause and cardiovascular mortality, regardless of SBP < 130 or > 140 mmHg as the reference group. On the other hand, low SBP (< 140 mmHg) combined with the low TyG index group was not statistically different, but a reduction in the risk of all-cause and cardiovascular mortality was still observed. This negative result did not reach statistical significance, presumably due to the insufficient sample size of this combined group. Therefore, future studies with larger cohorts are needed to confirm these findings.

Some potential mechanisms may explain the combined association of the TyG index and SBP with all-cause and cardiovascular mortality. Long-term hypertension can cause target organ damage, including heart, brain, and kidneys complications, resulting in an increased risk of mortality due to corresponding organ failure or cardiovascular and cerebrovascular events [40, 41]. The association of the TyG index with all-cause and cardiovascular mortality is mainly mediated through cardiovascular events [42]. First, the TyG index is calculated from FBG and TG, both of which are acknowledged risk factors for CVD [43, 44]. Elevated FBG levels can have toxic effects on heart tissue and blood vessels and cause disorders of lipid metabolism and abnormal platelet function [45]. In addition, hyperglycemic states have pro-inflammatory and cardiotoxic effects, which may adversely affect cardiovascular outcomes. Interestingly, Quagliariello V et al. [46] found that SGLT-2 inhibitors may reduce the adverse effects of inflammation and apoptosis through engaging NLRP3 and MyD88-related pathways in doxorubicin-treated mice, resulting in significant improvements in cardiac functions. Higher TG levels can cause disorders of lipid metabolism and atherosclerosis, leading to the development of CVD [47]. More importantly, higher levels of the TyG index reflect IR, which is related to the pathogenesis of CVD [48]. First, IR would cause elevated blood glucose levels, triggering inflammation, oxidative stress, and dyslipidemia, which can exacerbate CVD progression [49]. Second, IR induces abnormal secretion of nitric oxide (NO) and impairs endothelial function [50, 51]. Third, IR may exacerbate platelet aggregation and adhesion, promoting the formation of thrombosis and inflammation [52]. Fourth, insulin plays an important inhibitory role in the hydrolysis of triglycerides [53]. In the case of insufficient insulin secretion or insulin action failure, triglycerides' hydrolysis will be significantly accelerated, resulting in a large number of fatty acids entering the mitochondria for oxidation to supply energy [54]. In this process, excessive reactive oxygen species (ROS) are generated that would damage the mitochondrial function and reduce its viability [55]. The above factors may reasonably explain the mechanism of the role of the TyG index on the occurrence and development of CVD.

Some limitations of this study should be mentioned. First, the results of this study are from a cohort study. Although multivariate hazard regression models were used to adjust for confounding factors, the confounding effects on the results were not completely eliminated. In addition, some study individuals were excluded due to missing data and lost follow-up, which may introduce selection bias to the study results. Second, this study is a non-randomized controlled trial (RCT), and individuals with low SBP levels may not have been treated with antihypertensive medication and therefore may have failed to consider the clinical benefits of antihypertensive medication. Further RCTs are needed to validate these findings. Third, this study excluded those samples with missing data, which might result in a potential selection bias. Lastly, the findings were drawn from healthy individuals in the United States and may not apply to other regions or diseased populations.

## Conclusion

Our findings suggest that the combination of low TyG index and low SBP (< 120 mmHg and < 130 mmHg) was associated with a lower risk of all-cause and cardiovascular mortality. However, no survival benefit was observed in the combined group of low TyG index and SBP < 140 mmHg. Furthermore, the mortality rate in the combined group of low TyG index and low SBP gradually increased with the elevation of SBP level. This study's results may help clinicians identify potentially fatal risk factors as early as possible, thus effectively reducing the risk of cardiovascular events and death.

## Supplementary Information

**Additional file 1: Fig. S1.** Study population flow chart. **Fig. S2.** The mean (95% CI) of TyG index was 8.56 (8.52, 8.59). **Fig. S3.** The mean (95% CI) of SBP was 118.64 (117.89, 119.39) mmHg. **Fig. S4. **The correlation analysis between TyG index and SBP. **Table S1.** The correlation between TyG index and SBP was analyzed by Pearson correlation test. **Table S2.** Results of univariate Cox regression analysis. **Table S3.** ROC curve analysis determined optimal cut off thresholds for TyG and all-cause mortality. **Table S4.** ROC curve analysis determined optimal cut-off thresholds for TyG and cardiovascular mortality. **Table S5.** Univariate and multivariate Cox regression analysis of different SBP levels (<120 mmHg, 120-130 mmHg, and >130 mmHg) with all-cause and cardiovascular mortality**. Fig. S5. **Comparison of the predictive ability of SBP and TyG index for all-cause and cardiovascular mortality. **Table S6.** Univariate and multivariate Cox regression analysis of different SBP levels (<130 mmHg, 130-140 mmHg, and >140 mmHg) with all-cause and cardiovascular mortality.

## Data Availability

The NHANES datasets are available on DataDryad (https://doi.org/10.5061/dryad.d5h62).

## Abbreviations

CVD: Cardiovascular disease
DM: Diabetes mellitus
IR: Insulin resistance
CAD: Coronary artery disease
ACS: Acute coronary syndrome
HF: Heart failure
TyG: Triglyceride-glucose
SBP: Systolic blood pressure
ACEI: Angiotensin-converting enzyme inhibitors
ARB: Angiotensin receptor blocker
WC: Waist circumference
HR: Heart rate
MEC: Mobile examination center
BMI: Body mass index
TC: Total cholesterol
TG: Triglycerides
LDL-C: Low-density lipoprotein cholesterol
HDL-C: High-density lipoprotein cholesterol
FBG: Fasting blood glucose
HbA1c: Glycated haemoglobin
UA: Uric acid
eGFR: Estimated glomerular filtration rate
ROC: Receiver operating characteristic
HR: Hazard ratio
CI: Confidence interval
