# Supplementary material for: Combined association of triglyceride-glucose index and systolic blood pressure with all-cause and cardiovascular mortality among the general population
Source: J Transl Med. 2022 Oct 20;20:478. doi: 10.1186/s12967-022-03678-z (PMC9583494; doi:10.1186/s12967-022-03678-z)
Supplement: Supplementary file 1 — Additional file 1: Fig. S1. Study population flow chart. Fig. S2. The mean (95% CI) of TyG index was 8.56 (8.52, 8.59). Fig. S3. The mean (95% CI) of SBP was 118.64 (117.89, 119.39) mmHg. Fig. S4. The correlation analysis between TyG index and SBP. Table S1. The correlation between TyG index and SBP was analyzed by Pearson correlation test. Table S2. Results of univariate Cox regression analysis. Table S3. ROC curve analysis determined optimal cut off thresholds for TyG and all-cause mortality. Table S4. ROC curve analysis determined optimal cut-off thresholds for TyG and cardiovascular mortality. Table S5. Univariate and multivariate Cox regression analysis of different SBP levels (<120 mmHg, 120-130 mmHg, and >130 mmHg) with all-cause and cardiovascular mortality. Fig. S5. Comparison of the predictive ability of SBP and TyG index for all-cause and cardiovascular mortality. Table S6. Univariate and multivariate Cox regression analysis of different SBP levels (<130 mmHg, 130-140 mmHg, and >140 mmHg) with all-cause and cardiovascular mortality. [file 12967_2022_3678_MOESM1_ESM.docx]

**Figure S1.** Study population flow chart.


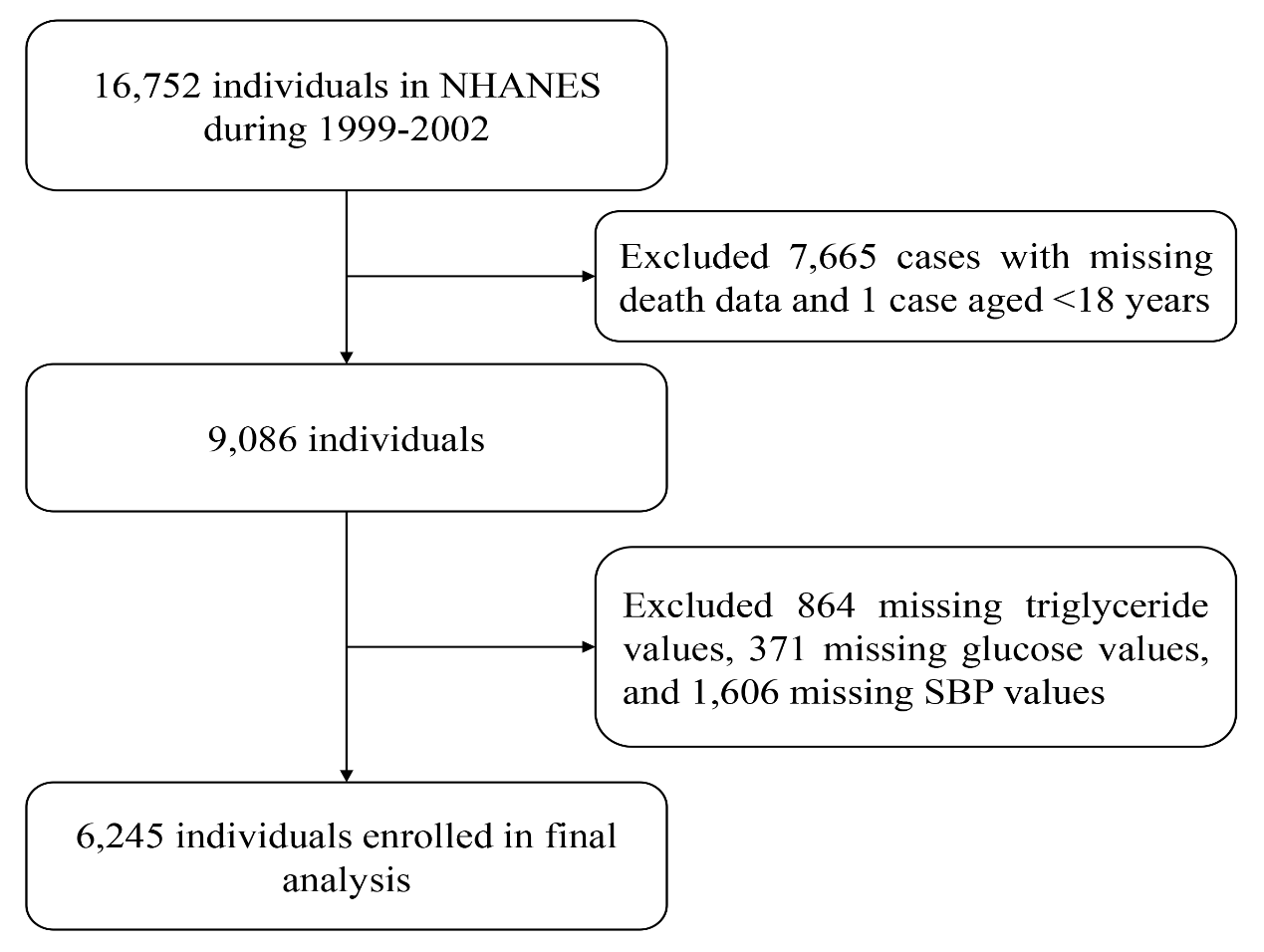


**Figure S2.** The mean (95% CI) of TyG index was 8.56 (8.52 ,8.59)


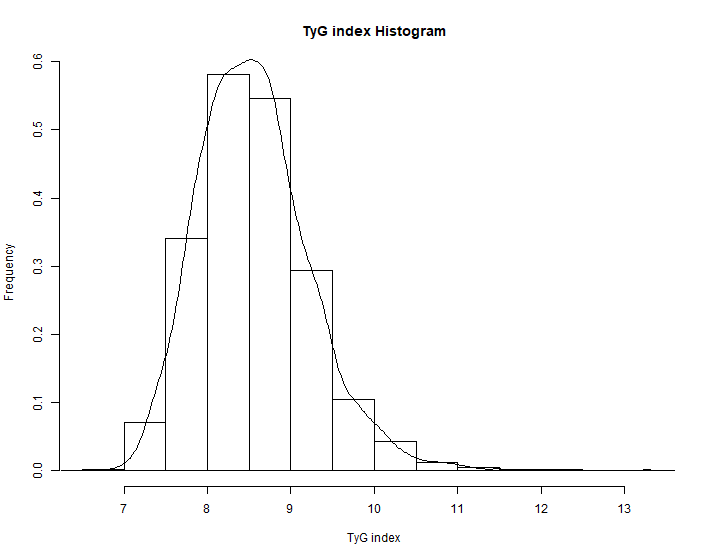


**Figure S3.** The mean (95% CI) of SBP was 118.64 (117.89 ,119.39) mmHg


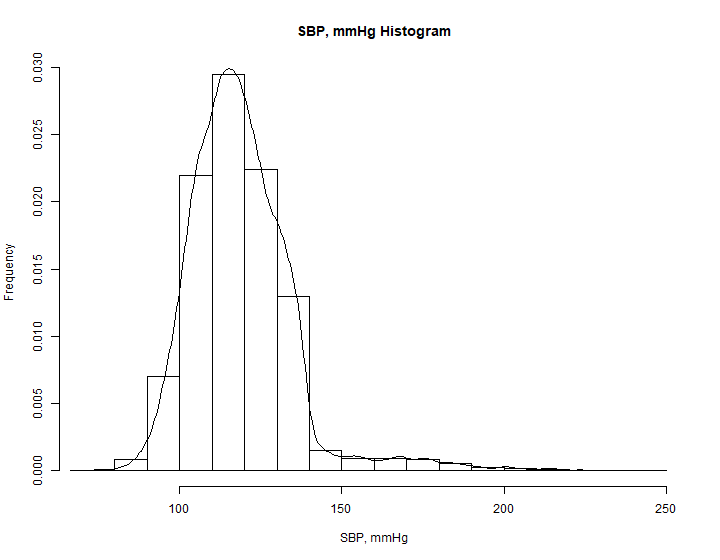


**Figure S4.** The correlation analysis between TyG index and SBP


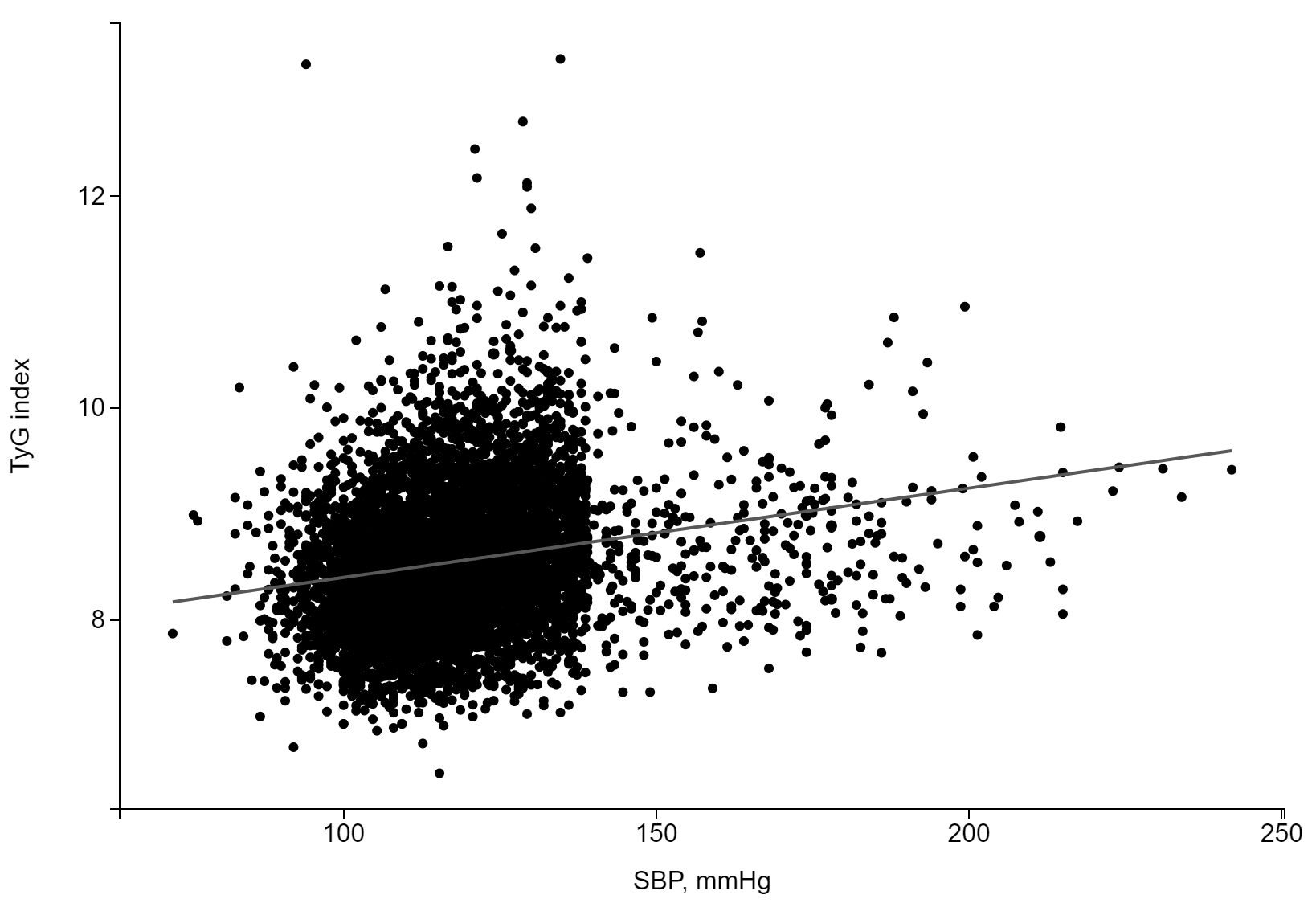


| **Table S1.** The correlation between TyG index and SBP was analyzed by Pearson correlation test | | | | | | |
| --- | --- | --- | --- | --- | --- | --- |
| Var 1 | Var 2 | Correlation | 95%CI low | 95%CI up | t | P.value |
| SBP, mmHg | TyG index | 0.2037 | 0.1798 | 0.2274 | 16.4413 | <0.0001 |

| **Table S2.** Results of univariate Cox regression analysis | | | | |
| --- | --- | --- | --- | --- |
| Variable | All‑cause mortality | | Cardiovascular mortality | |
|  | HR (95%CI) | P-value | HR (95%CI) P-value | |
| Age | 1.09 (1.08, 1.10) | <0.001 | 1.10 (1.08, 1.12) | <0.001 |
| Female | 0.62 (0.47, 0.82) | <0.001 | 0.58 (0.31, 1.08) | 0.146 |
| BMI | 0.99 (0.97, 1.02) | 0.675 | 0.91 (0.85, 0.98) | 0.054 |
| WC | 1.02 (1.01, 1.02) | 0.002 | 1.00 (0.97, 1.02) | 0.776 |
| SBP ^a^ | 1.17 (1.10, 1.25) | <0.001 | 1.25 (1.10, 1.42) | 0.007 |
| DBP ^b^ | 0.78 (0.71, 0.86) | <0.001 | 0.82 (0.66, 1.02) | 0.299 |
| HR ^c^ | 1.15 (1.04, 1.29) | 0.004 | 1.10 (0.86, 1.41) | 0.525 |
| Education |  |  |  |  |
| < High school | Ref. |  | Ref. |  |
| High school | 0.61 (0.43, 0.85) | <0.001 | 0.40 (0.19, 0.84) | 0.062 |
| > High school | 0.30 (0.22, 0.41) | <0.001 | 0.17 (0.08, 0.36) | <0.001 |
| Physical activity | |  |  |  |
| Sedentary | Ref. |  | Ref. |  |
| Low | 0.28 (0.20, 0.40) | <0.001 | 0.14 (0.05, 0.35) | <0.001 |
| Moderate | 0.12 (0.07, 0.22) | <0.001 | 0.11 (0.04, 0.35) | <0.0011 |
| Vigorous | 0.18 (0.12, 0.27) | <0.001 | 0.09 (0.03, 0.24) | <0.001 |
| Smoking | 2.58 (1.86, 3.58) | <0.001 | 1.52 (0.75, 3.07) | 0.208 |
| Drinking | 1.73 (1.23, 2.45) | 0.011 | 0.83 (0.31, 2.20) | 0.637 |
| Diabetes | 4.10 (2.89, 5.83) | <0.001 | 7.02 (3.55, 13.91) | <0.001 |
| Hypertension | 3.75 (2.85, 4.92) | <0.001 | 11.70 (5.82, 23.51) | <0.001 |
| CAD | 5.72 (3.80, 8.61) | <0.001 | 10.36 (4.94, 21.69) | <0.001 |
| FBG | 1.15 (1.11, 1.18) | <0.001 | 1.18 (1.12, 1.25) | <0.001 |
| HbA1c | 1.37 (1.29, 1.47) | <0.001 | 1.42 (1.24, 1.62) | <0.001 |
| TC | 1.30 (1.20, 1.42) | <0.001 | 1.22 (1.00, 1.51) | 0.048 |
| TG | 1.09 (1.06, 1.12) | <0.001 | 1.05 (0.94, 1.18) | 0.107 |
| LDL-C | 1.13 (0.98, 1.30) | 0.263 | 1.22 (0.90, 1.66) | 0.256 |
| HDL-C | 1.06 (0.75, 1.49) | 0.777 | 1.06 (0.50, 2.28) | 0.901 |
| UA ^d^ | 1.05 (1.04, 1.07) | <0.001 | 1.07 (1.04, 1.10) | 0.002 |
| Creatinine ^e^ | 1.05 (1.04, 1.06) | <0.001 | 1.05 (1.02, 1.07) | <0.001 |
| eGFR ^f^ | 1.02 (1.01, 1.02) | <0.001 | 1.02 (1.00, 1.03) | <0.0014 |
| TyG index | 1.85 (1.58, 2.18) | <0.001 | 1.78 (1.24, 2.56) | 0.015 |

^a^ indicates per 1-unit changed in SBP is 10 mmHg.

^b^ indicates per 1-unit changed in DBP is 10 mmHg.

^c^ indicates per 1-unit changed in HR is 10 bpm.

d indicates per 1-unit changed in UA is 10 umol/L.

e indicates per 1-unit changed in creatinine is 10 umol/L.

^f^ indicates per 1-unit changed in eGFR is 10 mL/min/1.73 m2.

BMI: body mass index; WC: waist circumference; SBP: systolic blood pressure; DBP: diastolic blood pressure; HR: heart rate; CAD: coronary atherosclerotic heart disease; FBG: fasting blood glucose; HbA1c: glycated haemoglobin; TC: total cholesterol; TG: triglycerides; LDL-C: low-density lipoprotein cholesterol; HDL-C: high-density lipoprotein cholesterol; UA: uric acid; eGFR: estimated glomerular filtration rate; TyG: triglyceride glucose; HR: Hazard ratio; CI: confidence interval.

| **Table S3.** ROC curve analysis determined optimal cut off thresholds for TyG and all-cause mortality | | | | | | |
| --- | --- | --- | --- | --- | --- | --- |
| Test | Best threshold | Sensitivity | Specificity | Predict.time (month) | Survival | ROC area (AUC) |
| TyG index | 8.86036 | 0.43817 | 0.71308 | 55 | 0.96504 | 0.58065 |

| **Table S4.** ROC curve analysis determined optimal cut-off thresholds for TyG and cardiovascular mortality | | | | | | |
| --- | --- | --- | --- | --- | --- | --- |
| Test | Best threshold | Sensitivity | Specificity | Predict.time (month) | Survival | ROC area (AUC) |
| TyG index | 8.55188 | 0.56549 | 0.5278 | 53 | 0.99247 | 0.54148 |

| **Table S5.** Univariate and multivariate Cox regression analysis of different SBP levels (<120 mmHg, 120-130 mmHg, and >130 mmHg) with all-cause and cardiovascular mortality | | | | | |
| --- | --- | --- | --- | --- | --- |
| Variables | Mortality Rate (per 100000 person-years) | Univariate analysis |  | Multivariate analysis | |
|  |  | HR (95% CI) | P-value | HR (95% CI) | P-value |
| **All-cause mortality** |  |  |  |  |  |
| SBP category |  |  |  |  |  |
| <120 mmHg | 209 | Ref. |  | Ref. |  |
| 120-130 mmHg | 417 | 1.88 (1.34, 2.64) | 0.001 | 1.85 (1.28, 2.69) | 0.004 |
| >130 mmHg | 578 | 2.69 (1.68, 4.31) | 0.001 | 2.08 (1.22, 3.54) | 0.015 |
| SBP category |  |  |  |  |  |
| >130 mmHg | 578 | Ref. |  | Ref. |  |
| 120-130 mmHg | 417 | 0.70 (0.46, 1.06) | 0.102 | 0.89 (0.56, 1.43) | 0.638 |
| <120 mmHg | 209 | 0.37 (0.23, 0.60) | 0.102 | 0.48 (0.28, 0.82) | 0.015 |
| **Cardiovascular mortality** | |  |  |  |  |
| SBP category |  |  |  |  |  |
| <120 mmHg | 20 | Ref. |  | Ref. |  |
| 120-130 mmHg | 71 | 2.23 (1.13, 4.40) | 0.029 | 2.23 (1.11, 4.48) | 0.034 |
| >130 mmHg | 105 | 3.16 (1.46, 6.82) | 0.007 | 2.83 (1.27, 6.31) | 0.017 |
| SBP category |  |  |  |  |  |
| >130 mmHg | 105 | Ref. |  | Ref. |  |
| 120-130 mmHg | 71 | 0.71 (0.33, 1.49) | 0.369 | 0.79 (0.38, 1.63) | 0.524 |
| <120 mmHg | 20 | 0.32 (0.15, 0.68) | 0.007 | 0.35 (0.16, 0.79) | 0.017 |

Model adjusted for age, gender, BMI, physical activity, HR, current smoker, diabetes, antihypertensive drugs, glucose-lowering drugs, LDL-C, HDL-C, UA, eGFR.

**Figure S5.** Comparison of the predictive ability of SBP and TyG index for all-cause and cardiovascular mortality


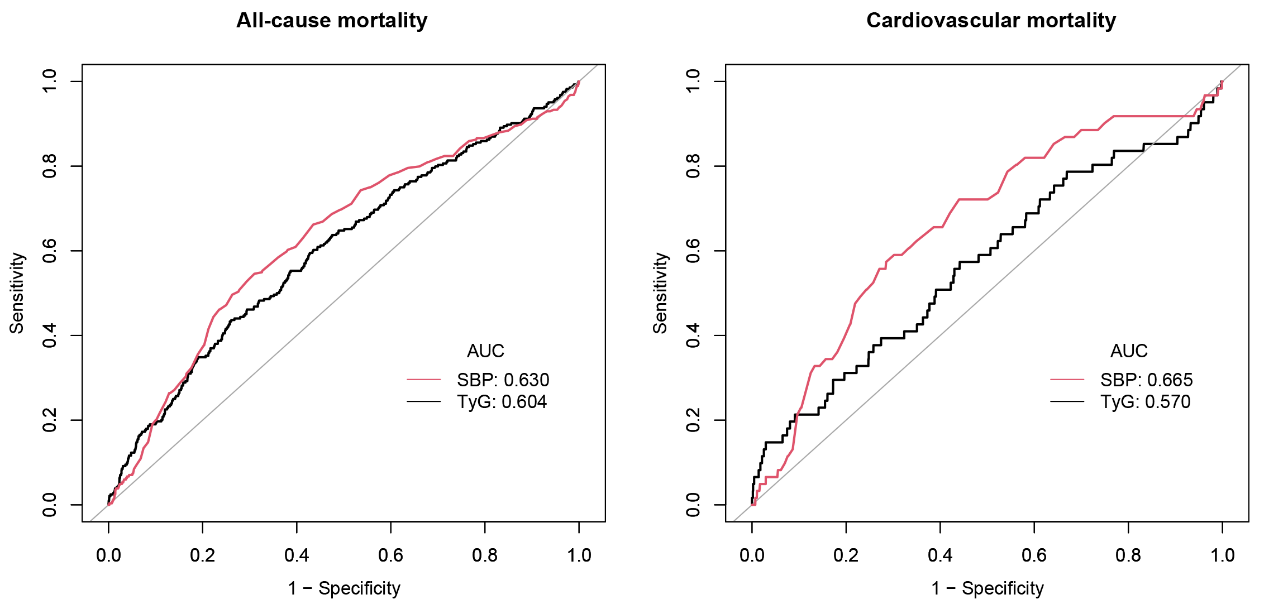


| **Table S6.** Univariate and multivariate Cox regression analysis of different SBP levels (<130 mmHg, 130-140 mmHg, and >140 mmHg) with all-cause and cardiovascular mortality | | | | | |
| --- | --- | --- | --- | --- | --- |
| Variables | Mortality Rate (per 100000 person-years) | Univariate analysis |  | Multivariate analysis | |
|  |  | HR (95% CI) | P-value | HR (95% CI) | P-value |
| **All-cause mortality** | |  |  |  |  |
| SBP category |  |  |  |  |  |
| <130 mmHg | 276 | Ref. |  | Ref. |  |
| 130-140 mmHg | 624 | 2.32 (1.60, 3.35) | <0.001 | 1.72 (1.08, 2.73) | 0.033 |
| >140 mmHg | 512 | 1.81 (0.90, 3.61) | 0.106 | 1.41 (0.66, 3.02) | 0.383 |
| SBP category |  |  |  |  |  |
| >140 mmHg | 512 | Ref. |  | Ref. |  |
| 130-140 mmHg | 624 | 1.28 (0.77, 2.13) | 0.347 | 1.22 (0.64, 2.32) | 0.560 |
| <130 mmHg | 276 | 0.55 (0.28, 1.11) | 0.106 | 0.71 (0.33, 1.51) | 0.383 |
| **Cardiovascular mortality** | |  |  |  |  |
| SBP category |  |  |  |  |  |
| <130 mmHg | 41 | Ref. |  | Ref. |  |
| 130-140 mmHg | 127 | 2.36 (1.10, 5.07) | 0.037 | 2.27 (0.98, 4.36) | 0.069 |
| >140 mmHg | 104 | 2.21 (0.64, 8.60) | 0.211 | 2.19 (0.57, 8.41) | 0.265 |
| SBP category |  |  |  |  |  |
| >140 mmHg | 104 | Ref. |  | Ref. |  |
| 130-140 mmHg | 127 | 1.11 (0.23, 4.39) | 0.992 | 1.08 (0.21, 4.18) | 0.941 |
| <130 mmHg | 41 | 0.43 (0.12, 1.57) | 0.211 | 0.46 (0.12, 1.76) | 0.265 |

Model adjusted for age, gender, BMI, physical activity, HR, current smoker, diabetes, antihypertensive drugs, glucose-lowering drugs, LDL-C, HDL-C, UA, eGFR.
